# Crystal structure and biochemical activity of the macrodomain from rubella virus p150

**DOI:** 10.1128/jvi.01777-23

**Published:** 2024-01-30

**Authors:** Guido A. Stoll, Nikos Nikolopoulos, Haoming Zhai, Liao Zhang, Christopher H. Douse, Yorgo Modis

**Affiliations:** a Molecular Immunity Unit, Department of Medicine, University of Cambridge, MRC Laboratory of Molecular Biology, Cambridge CB2 0QH, UK; b Cambridge Institute of Therapeutic Immunology & Infectious Disease (CITIID), Department of Medicine, University of Cambridge, Cambridge CB2 0AW, UK; c Department of Experimental Medical Science, Lund University, Sölvegatan 17, 221 84 Lund, Sweden

**Keywords:** macrodomain, ADP-ribose, mono-ADP-ribosylhydrolase, de-MARylation, rubella virus, de-MARylase, ADP ribosylation, enzymes, X-ray crystallography, alphavirus

## Abstract

Rubella virus encodes a nonstructural polyprotein with RNA polymerase, methyltransferase, and papain-like cysteine protease activities, along with a putative macrodomain of unknown function. Macrodomains bind ADP-ribose adducts, a post-translational modification that plays a key role in host-virus conflicts. Some macrodomains can also remove the mono-ADP-ribose adduct or degrade poly-ADP-ribose chains. Here, we report high-resolution crystal structures of the macrodomain from rubella virus nonstructural protein p150, with and without ADP-ribose bound. The overall fold is most similar to macroD-type macrodomains from various nonviral species. The specific composition and structure of the residues that coordinate ADP-ribose in the rubella virus macrodomain are most similar to those of macrodomains from alphaviruses. Isothermal calorimetry shows that the rubella virus macrodomain binds ADP-ribose in solution. Enzyme assays show that the rubella virus macrodomain can hydrolyze both mono- and poly-ADP-ribose adducts. Site-directed mutagenesis identifies Asn39 and Cys49 and required for mono-ADP-ribosylhydrolase (de-MARylation) activity.

**Importance:**

Rubella virus remains a global health threat. Rubella infections during pregnancy can cause serious congenital pathology and no antiviral treatments are available. Our work demonstrates that, like alpha- and coronaviruses, rubiviruses encode a mono-ADP-ribosylhydrolase with a structurally conserved macrodomain fold to counteract MARylation by PARPs in the host innate immune response. Our structural data will guide future efforts to develop novel antiviral therapeutics against rubella or infections with related viruses.

## Introduction

Rubella virus (RuV), from the *Rubivirus* genus (family *Matonaviridae*), causes rubella, also known as German measles. Rubella is usually a mild disease but infection in the first trimester of pregnancy can result in miscarriage or infants with congenital rubella syndrome (CRS) (1). CRS symptoms include cataracts, deafness, and congenital hearth or brain defects. Rubella virus only infects humans, via the respiratory route, but two recently discovered rubiviruses, ruhugu and rustrela viruses, infect multiple other mammalian species (2). A single vaccine dose, usually as part of the measles, mumps, and rubella (MMR) vaccine, is 95% effective to prevent rubella, but no antiviral treatments are available (1). Rubella remains a common infection worldwide, with 100,000 annual CRS cases (1).

Rubella virus is a member of the alphavirus supergroup, referred to since 2019 as the *Alsuviricetes* class (3), which includes rubiviruses, alphaviruses, hepatitis E virus, and nine other families of viruses that mostly infect plants. Rubella virus is a positive-strand enveloped RNA virus that forms pleiomorphic virions 50-90 nm in length (4, 5). Rubella virus has a 10-kilobase RNA genome with exceptionally high GC content (70%) (4, 6). The virus encodes two polyproteins, one structural and one nonstructural (4). The nonstructural protein, p200, is autoproteolytically cleaved early in infection into the products necessary for viral replication, the p150 and p90 proteins (7). p90 contains the helicase and RNA-dependent RNA polymerase (RdRp) activities required for genome replication (8). p150 contains a methyltransferase domain and a papain-like cysteine protease domain (9, 10). The p150 papain-like protease in has a similar overall structure and catalytic core as the papain-like proteases from SARS-CoV-2 and foot-and-mouth disease virus (10). The papain-like protease is the only protease encoded by rubella virus and is responsible for processing of the viral polyprotein (4, 7, 9).

Coronaviruses and a subset of *Alsuviricetes* that infect animals contain papain-like proteases flanked by a macrodomain (9, 11). Macrodomains bind ADP-ribose, an important post-translational modification, and some of its metabolites or derivatives (12). Macrodomains may catalyze the removal of the mono-ADP-ribose (MAR) adduct, usually to a glutamate or aspartate residue, or the degradation of poly-ADP-ribose (PAR) chains (13, 14). Crystal structures of the macrodomains from alpha- and coronaviruses, including SARS-CoV-2, identify the ADP-ribose binding pockets of viral macrodomains (15–26). The conserved macrodomains from alphaviruses, coronaviruses, and hepatitis E virus are mono-ADP-ribosylhydrolases, or de-MARylases (15, 23–28). Some viral macrodomains can also hydrolyze PAR chains, albeit inefficiently (15, 27). Most of the conserved residues that coordinate ADP-ribose in viral macrodomains are required for de-MARylase activity (15). The macrodomain from mouse hepatitis virus (MHV), a coronavirus, is responsible for liver pathology and mutations in the MHV macrodomain attenuate virus replication (29, 30). The de-MARylation activity of alphavirus macrodomains promotes virus replication by preventing stress-granule formation (31). Similarly, the conserved macrodomain (Mac1) from SARS-CoV promotes virulence and suppresses IFN-β induction (32). Conversely, MARylation of aspartate, glutamate, and lysine side chains by a set of MARylases from the PARP family, including PARP10 and PARP14, inhibits translation and alphavirus replication (15, 33–36). These PARPs are interferon-stimulated genes (ISGs) (33) and are evolving rapidly under strong recurrent positive selection (37). Addition and removal of MARylation by cellular PARP catalytic domains and viral macrodomains, respectively, is therefore an important component of host-virus conflict (15, 34, 36, 37). Viral macrodomains and their active sites are proving to be attractive targets for antiviral therapeutics, as illustrated by multiple recent high-throughput screens identifying small molecules that target SARS-CoV-2 Mac1, including a structure-based study (38–40).

The structure and biochemical properties of the putative macrodomain from rubella virus remain unknown. Here, we report high-resolution crystal structures of the rubella virus p150 macrodomain, with and without ADP-ribose bound. The overall fold is most similar to macroD-type macrodomains from various viral and nonviral species. The detailed composition and structure of the residues poised for catalysis or coordinating ADP-ribose in the rubella virus macrodomain are most similar to those of macrodomains from alphaviruses. We show that the rubella virus macrodomain binds ADP-ribose in solution and has both de-MARylation activity and de-PARylation activity. Asparagine and cysteine residues (Cys39 and Asn49 in the macro domain, corresponding to 841 and 851 in p150) contribute to catalysis. Our work demonstrates that, like alpha- and coronaviruses, rubiviruses encode an ADP-ribosyl hydrolase with a structurally conserved macrodomain fold to counteract MARylation by PARPs in the host innate immune response. Our structural data will guide future efforts to develop novel antiviral therapeutics against rubella or infections with related viruses.

## Results

### Crystal structure of the RuV p150 macrodomain

**Fig. 1:**
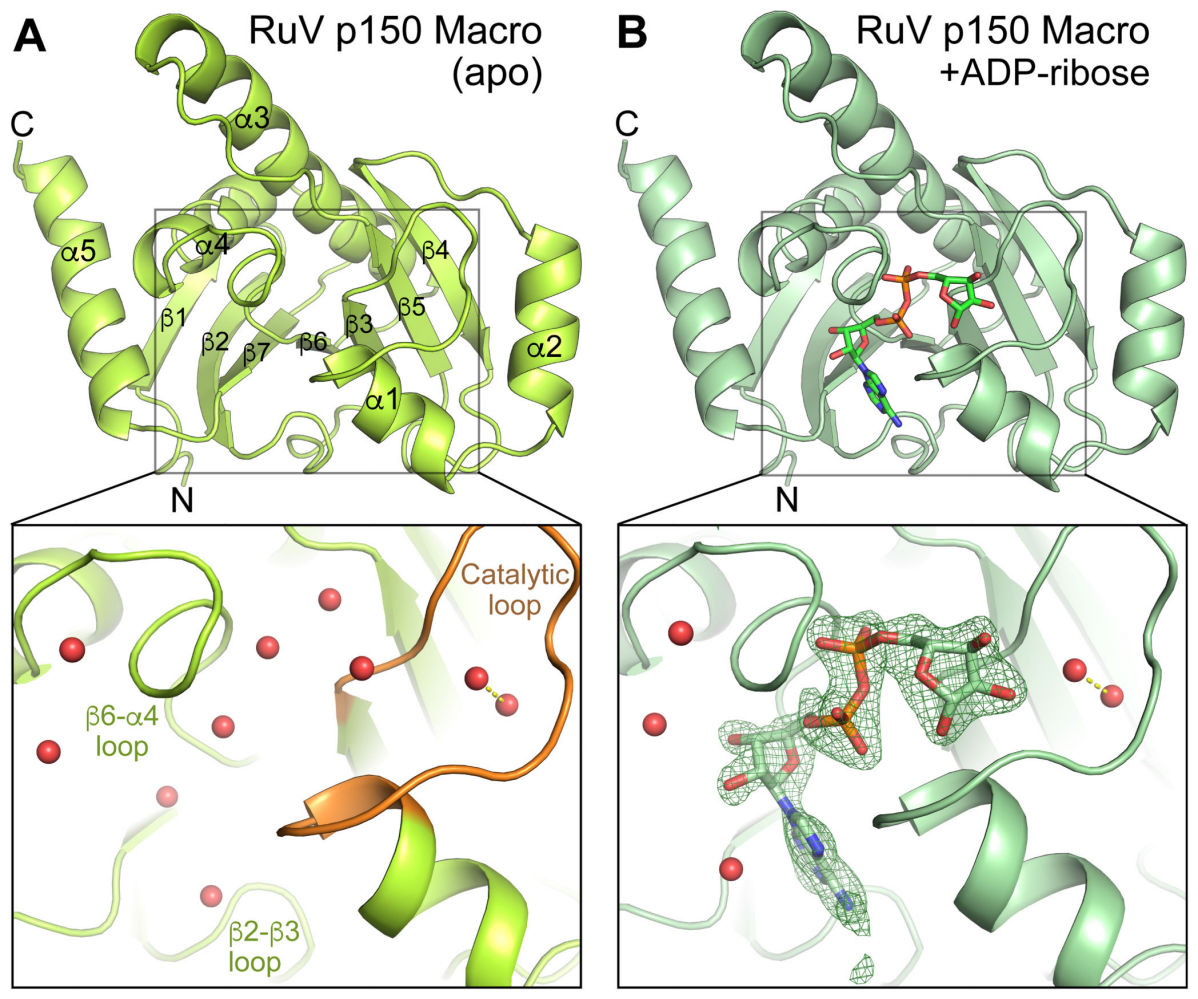
Crystal structures of the macrodomain from rubella virus (RuV) p150. **(A)** Structure of RuV macrodomain alone, with the catalytic loop (β3-α1 loop) in orange. Red, water molecules. Dashed line, hydrogen bond. The three substrate-coordinate loops are labeled. **(B)** Structure of RuV macrodomain with ADP-ribose (ADPr) bound. Closeup panels show ADPr binding pocket. The green mesh is a polder map calculated with the ADPr molecule omitted (more specifically, an *F*_obs_ – *F*_calc_ Fourier difference map was calculated with ADPr omitted from the model and the bulk solvent excluded from the omitted region, as implemented in Phenix (52)). An isomesh contour level of 4.2 σ in PyMol (Schrödinger, LLC) was used. See Table 1 for crystallographic data collection, refinement, and validation statistics.

We first determined the crystal structure of the macrodomain from rubella virus p150 (RuV macrodomain), spanning residues 805-983 of p150 (UniProt G3M8F4). The structure was determined at 1.7 Å resolution by automated molecular replacement with MrBUMP (41, 42) using an atomic search model based on the human protein-proximal ADP-ribosyl-hydrolase MacroD2 structure (PDB 4IQY) (43). See Table 1 for crystallographic data collection, refinement, and validation statistics. The overall fold is typical for a macrodomain: a central 7-stranded β-sheet surrounded by five peripheral α-helices (Fig. 1A). The most structurally similar proteins identified with DALI (44) were macrodomains from nonviral species including bacteria, archaea and vertebrates: *Oceanobacillus iheyensis* macroD (Z-score 23.7, rmsd 2.6 Å, PDB 5LAU and 5L9K (45)); *Archaeoglobus fulgidus* AF1521 macrodomain (Z-score 23.7, rmsd 2.2 Å, PDB 1HJZ (46) and 2BFQ (12)); and human PARP14 macro 1 (Z-score 23.5, rmsd 2.1 Å, PDB 3Q6Z (35)). However, the overall fold of RuV macrodomain also closely resembles those of macrodomains from coronaviruses and alphaviruses, for example SARS-CoV-2 Mac1 (Z-score 21.0-21.6, rmsd 2.2-2.3 Å, PDB 6WOJ, 6W02, 6YWL and 7TX5 (20, 21, 23, 24)) and Getah virus macrodomain (Z-score 20.5, rmsd 2.6 Å, PDB 6R0F and 6R0G (22)).

### Structure of RuV macrodomain with ADP-ribose bound

**Fig. 2:**
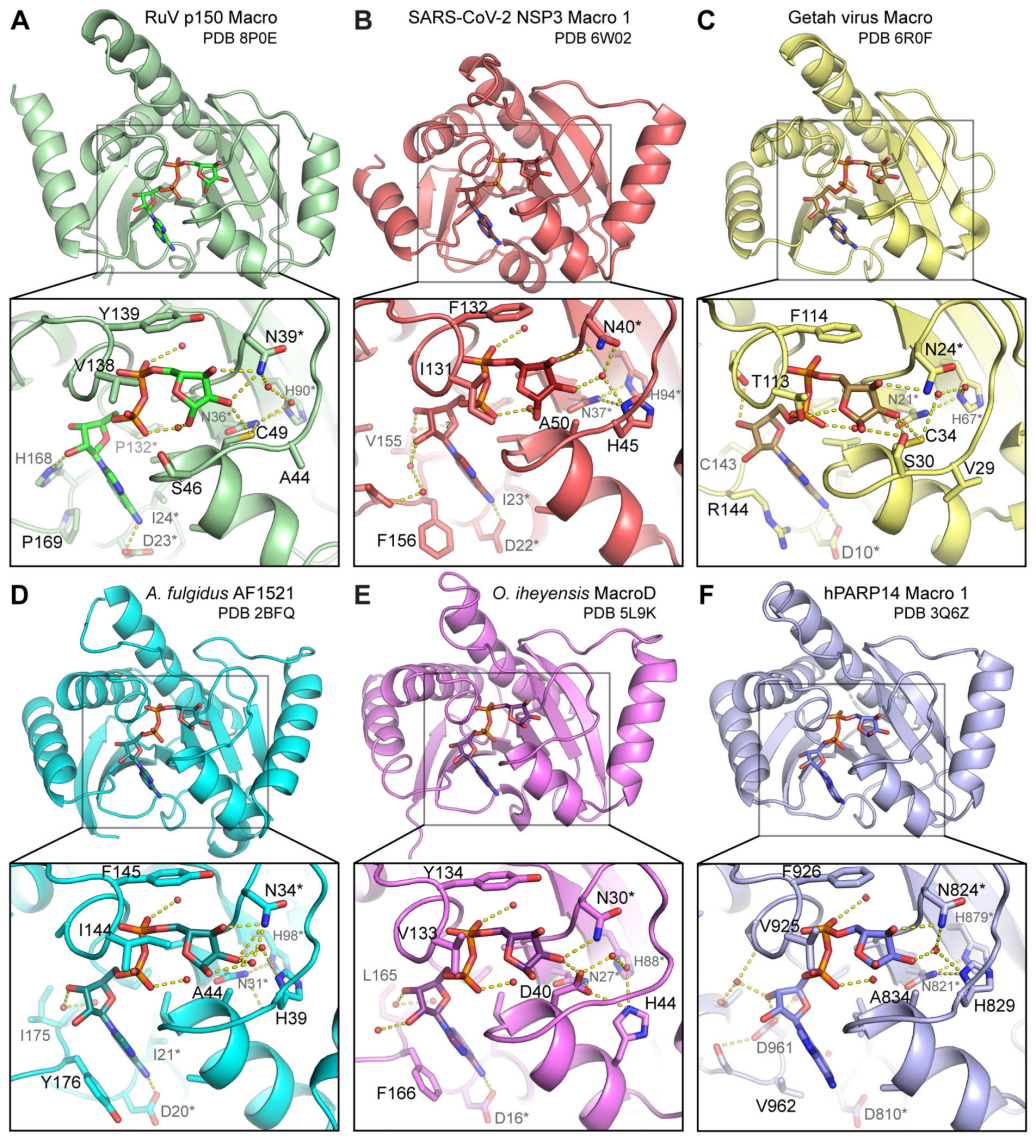
Comparison of the RuV macrodomain structure to macrodomains with similar structures. Overviews and closeups of the ADP-ribose (ADPr) binding pockets of: (**A**) RuV macrodomain, (**B**) SARS-CoV-2 NSP3 Mac1, (**C**) Getah virus macrodomain, (**D**) *Archaeoglobus fulgidus* AF1521 macrodomain, **(E**) *Oceanobacillus iheyensis* macroD, (**F**) human PARP14 macro 1. Side chains interacting with ADPr are shown; asterisks denote conserved residues. Dashed lines represent hydrogen bonds.

**Fig. 3:**
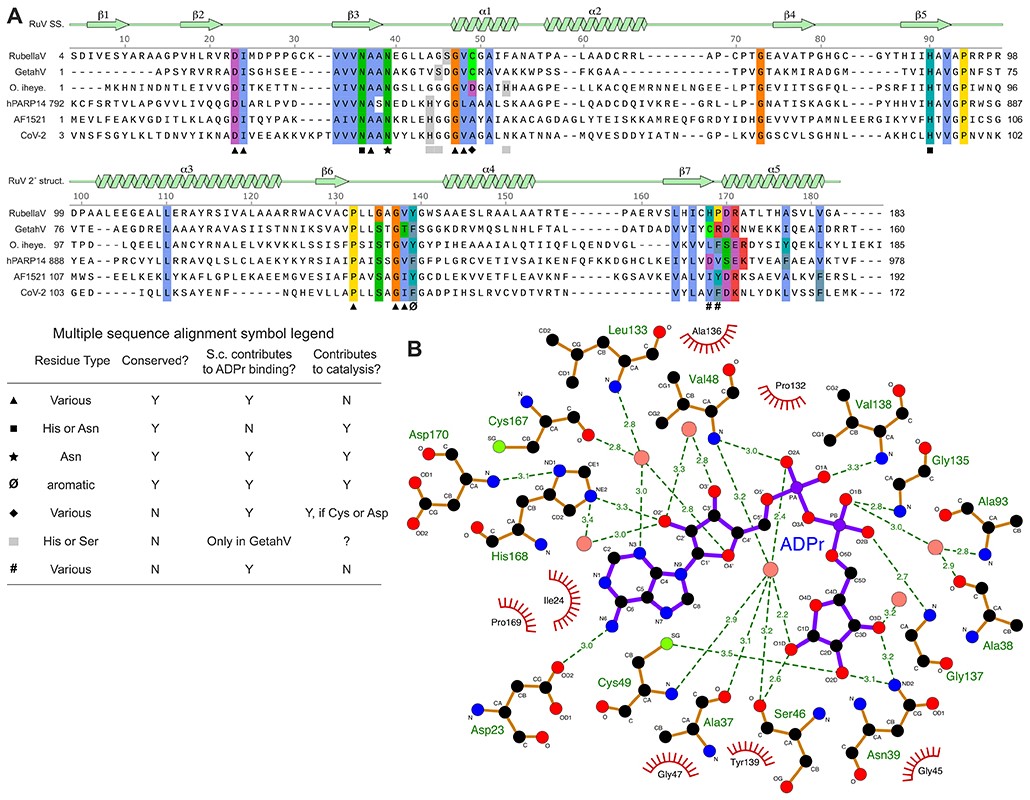
Sequence conservation and interatomic contacts in the RuV macrodomain ADP-ribose (ADPr) binding pocket. (**A**) Structure-based protein sequence alignment of RuV macrodomain and the structurally similar macrodomains shown in Fig. 2: Getah virus macrodomain (GetahV), *O. iheyensis* macroD (O. iheye.), human PARP14 macro 1 (hPARP14), *A. fulgidus* AF1521 macrodomain, and SARS-CoV-2 NSP3 Mac1 (CoV-2). The secondary structure of RuV macrodomain is shown above. Residue numbers above the alignment refer to RuV macrodomain (add 802 to get p150 residue numbers). Residues forming interactions with ADPr and selected conserved residues near the ADPr binding pocket are colored by residue type. Residues forming key contacts with ADPr or potentially contributing to catalysis are denoted with black symbols – see table (lower left) for legend. (**B**) Schematic of the RuV macrodomain residues that form hydrogen bonds (green dashes, with bond length in Å) and hydrophobic contacts (red arcs) with ADPr. Pink, water molecules. S.c., side chain. Based on output from LigPlot+ v.2.2.8 (54).

Since the macrodomains with the most similar structures to RuV macrodomain could all be crystallized with ADP-ribose bound, we soaked RuV macrodomain crystals in a solution containing 2 mM ADP-ribose and hence obtained a structure with ADP-ribose bound. The ADP-ribose molecule is in in the β-anomeric configuration and binds to RuV macrodomain in a similar manner to other macrodomains (Fig. 1B). The binding pocket is formed primarily by the β3-α1 loop, also known as the catalytic loop or Loop 1, the β6-α4 loop, and the β2-β3 loop. The ADP-ribose molecule forms two hydrogen bonds and several hydrophobic contacts with side chains that are broadly conserved in macrodomains from different viral, bacterial, and vertebrate species (Figs. 2 and 3). One of the conserved hydrogen bonds is between an aspartate in the β2-β3 loop (Asp23 in RuV macrodomain) and the amide group in the adenine moiety of ADP-ribose (Fig. 2A). The second hydrogen bond is between the second asparagine in the catalytic loop (Asn39 in RuV macrodomain, Asn841 in RuV p150) and the 3’ or 2’ oxygen atom in the ribosyl (distal ribose) moiety of ADP-ribose. The hydrophobic contacts are with conserved aliphatic side chains in all three of the substrate-coordinating loops. In RuV macrodomain, these aliphatic residues are Ile24 in the β2-β3 loop, Ala37 and Val48 in the catalytic loop, and Pro132 and Val138 in the β6-α4 loop (Fig. 2A). Additionally, a conserved aromatic side chain in the β6-α4 loop (Tyr139 in RuV macrodomain) forms a hydrophobic contact with the 4’ and 5’ carbon atoms in the distal ribose. Also conserved are three water molecules directly coordinating the ADP-ribose molecule, forming hydrogen bonds with the α- and β-phosphate, the distal ribose, and multiple residues the catalytic loop (Fig. 2A-F). One of these water molecules, positioned between the α-phosphate and distal ribose, has been proposed to mediate substrate-assisted catalysis in human MacroD2 (43) (see below). The conserved residues and water molecules involved in ADP-ribose binding in the RuV macrodomain structure, and the interatomic contacts they form are shown in Fig. 3.

In addition to the contacts with conserved side chains listed above, multiple hydrogen bonds with main chain atoms of residues in all three substrate-coordinating loops contribute to ADP-ribose binding (Fig. 3). Many of these residues (residues 24, 46, 48, and 135-139 in RuV macrodomain) lack sequence conservation, consistent with the ligand contacts involving main chain atoms. Furthermore, unconserved side chain residues in the β7-α5 loop of macrodomains from different species (residues 168-169 in RuV macrodomain) form various types of contacts with ADP-ribose, including hydrogen bonds, hydrophobic interactions, and π-stacking interactions with the adenosine moiety of ADP-ribose (Fig. 2A-F).

### Conserved and novel features in the putative RuV macrodomain active site region

The ADP-ribose binding pocket is broadly conserved in macrodomains but there are differences in the composition and structure of the active sites of macrodomains from different classes or with different catalytic activities. The closest structural homologs of RuV macrodomain are macroD-type de-MARylases that remove ADP-ribosylation of glutamate or aspartate residues, namely *O. iheyensis* macroD (45), *A. fulgidus* AF1521 macrodomain (46), SARS-CoV-2 Mac1, and Getah virus macrodomain (22) (Fig. 2A-E). The defining active site signatures shared by these and other macroD-type domains are Nx(6)GG[V/L/I] and G[V/I/A][Y/F]G motifs in the catalytic and β6-α4 loops, respectively, and water molecules with catalytic potential at conserved positions coordinating the α- and β-phosphate groups (13, 43, 45). The human PARP14 macro 1 domain, though previously classified as macroH2A-like (13), contains all the macroD active site signatures and has comparable structural homology to RuV macrodomain (Fig. 2F). The putative active site of RuV macrodomain contains all of the macroD-type signatures including the catalytic asparagine, Asn39 in RuV macrodomain (Fig. 2A), except for a serine substitution in the first macroD motif (serine at position 46 instead of a glycine, see also below). We show below that mutation of Asn39 reduces the de-MARylation activity of RuV macrodomain to 20% of wild type. The RuV macrodomain active site lacks lysine and glutamate residues, which promote poly-ADP ribose hydrolysis in macrodomains from the ALC1-like (TARG1) and PARG-like classes, respectively (14, 47). Thus, based on the sequence and structure of RuV macrodomain active site residues, we conclude that RuV macrodomain is a member of the macroD-type class.

Although RuV macrodomain contains the macroD-type active site signatures, some atypical substitutions are present in the putative active site. Most notably, RuV macrodomain has a cysteine in the catalytic loop, at position 49 at the N-terminal end of helix α1 (Figs. 2A and 3A). An aspartate is found at this position in some of the closest macroD homologs, including *O. iheyensis* macroD, in which the aspartate (Asp40) contributes to substrate binding and catalysis (Figs. 2E and 3A) (45). However, an aspartate at this position is not absolutely required for de-MARylation activity (43, 45). A cysteine is also present at the equivalent position in the other rubiviruses (ruhugu and rustrella viruses), alphaviruses (25, 26), and in a minority fraction of the most similar protein sequences to RuV macrodomain, mostly from microbiotal species of bacteria. These bacterial species include high GC Gram-positive bacteria such as *Bifidobacterium longum*, which is worth noting given the exceptionally high GC content of the rubella virus genome (4, 6). Crystal structures of the macrodomain from Getah virus, from the alphavirus genus, show that the equivalent cysteine (Cys34 in Getah virus) can form a covalent adduct with ADP-ribose (22). In the RuV macrodomain structure, the Cys49 sulfhydryl forms hydrogen bonds with ADP-ribose and a conserved structured water molecule (Fig. 2A). Together, the substrate interactions of Cys34 in Getah virus macro and Cys49 in RuV macrodomain suggest that a cysteine in this position in the catalytic loop may contribute directly to catalysis (22) in the macrodomains of alpha- and rubiviruses. Indeed, we show below that mutation Cys49 reduces and alters the kinetics of de-MARylation by RuV macrodomain.

Another notable atypical substitution in the catalytic loop of RuV macrodomain is a serine at residue 46 instead of a glycine in the first macroD signature motif, resulting in the sequence Nx(6)**S**_**46**_GV instead of the canonical Nx(6)GGV (Fig. 3A). Similarly, Getah virus macro has an unusual serine substitution in the preceding position, Ser30. The side chain of Ser30 forms contributes significantly to ADP-ribose binding, forming two hydrogen bonds with the distal ribose (Fig. 2C) (22). The Ser46 side chain in RuV macrodomain is similarly oriented, pointing towards the distal ribose of the bound ADP-ribose (Fig. 2A), although the minimum distance between the hydroxyls of the serine and the distal ribose, at 4.5 Å, is greater in RuV macrodomain than in Getah virus macro (2.8 to 3 Å). Overall, in comparison to the available ADP-ribose-bound macrodomain structures we conclude that the RuV macrodomain structure is most similar in its putative active site composition and organization to the structures of alphavirus macrodomains.

### ADP-ribose specifically binds and stabilizes RuV macrodomain in solution

**Fig. 4:**
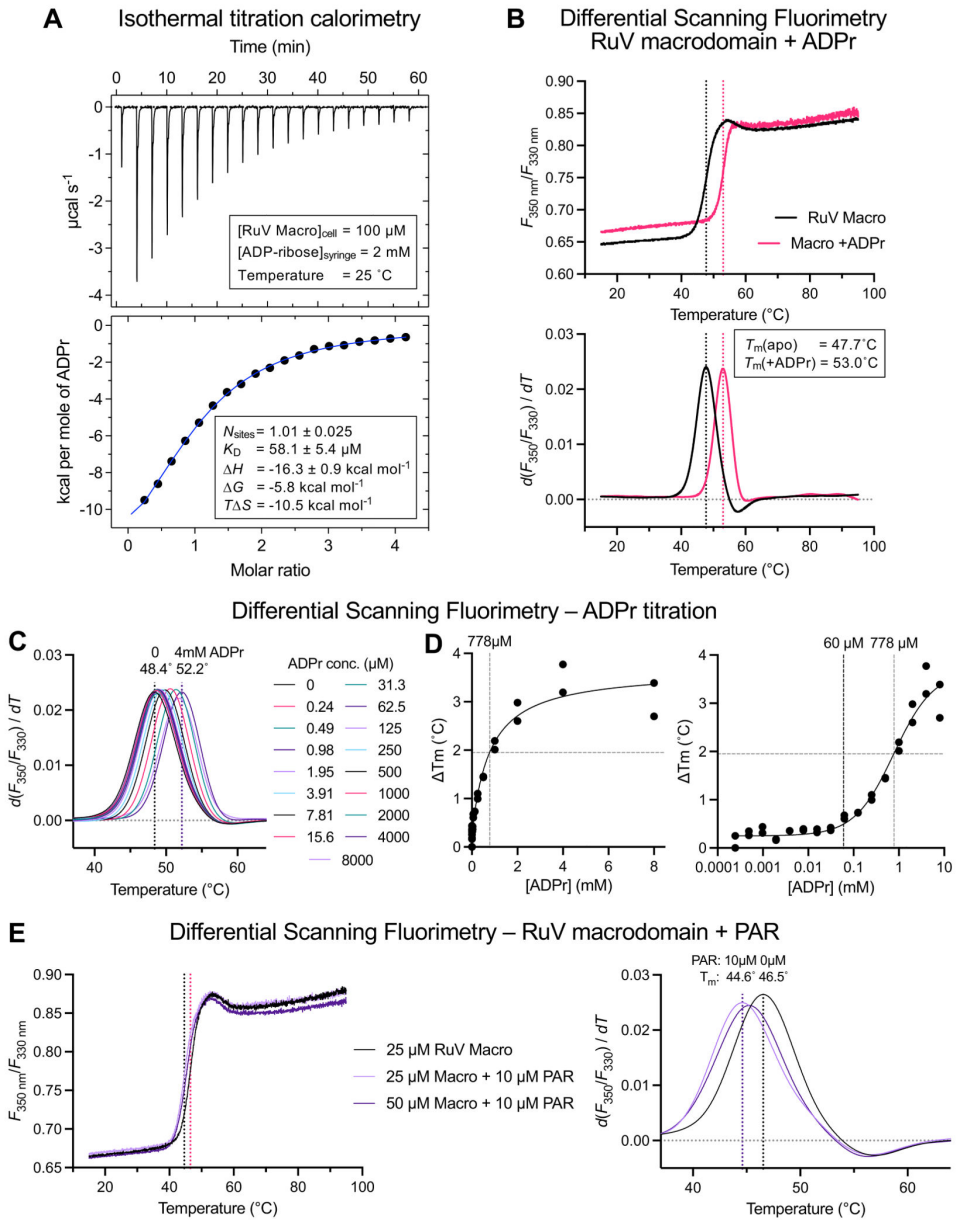
ADP-ribose (ADPr) and poly-ADP ribose (PAR) binding assays. (**A**) Isothermal titration calorimetry (ITC) binding assay with RuV macrodomain in the sample cell and ADPr as the injectant. (**B**) Differential scanning fluorimetry (DSF) of 25 µM RuV macrodomain with and without 5 mM ADPr. Intrinsic protein fluorescence at 330 nm and 350 nm was measured and the fluorescence ratio plotted as a function of temperature. The temperature value at the peak of the first derivative of the fluorescence ratio versus temperature function was taken as the melting temperature (T_m_). (**C**) DSF of 25 µM RuV macrodomain with titration of ADPr from 0 to 8 mM. (**D**) Plots of the change in T_m_ as a function of ADPr concentration, derived from the curves in (C) and a duplicate experiment. (**E**) DSF of RuV macrodomain with and without PAR.

We have shown above that the structure and ADP-ribose binding mode of RuV macrodomain are similar to those of other catalytically active macroD-type domains, but the ADP-ribose binding affinity and catalytic activity of RuV macrodomain in solution remain unknown. We therefore performed solution-based ligand binding assays with RuV macrodomain and ADP-ribose. Isothermal titration calorimetry (ITC) showed that RuV macrodomain binds ADP-ribose with a dissociation constant (*K*_D_) of 58.1 ± 5.4 µM (Fig. 4A). The ADPr binding affinity of RuV macrodomain is somewhat weaker than that of other viral macrodomains, for which the *K*_D_s are mostly in the range of 10 to 50 µM, and markedly weaker than the structurally similar nonviral *A. fulgidus* AF1521 macro, for which the *K*_D_ is 126 nM (Table 2) (12). The shape of the isotherm curve was indicative of specific, saturable binding. As expected from the structure of RuV macrodomain in complex with ADP-ribose, the protein:ADP-ribose binding stoichiometry was 1:1. Moreover, differential scanning fluorimetry (DSF) using the intrinsic fluorescence of RuV macrodomain as the readout revealed a clear thermal stabilization of the protein on binding ADP-ribose, with an increase in melting temperature of up to 5.3˚C upon saturation of the ADP-ribose binding site (Fig. 4B). Titration of ADPr via sequential twofold dilutions from 8 mM to 0.24 µM showed that 60 µM was the minimum ADPr concentration required to increase the melting temperature of RuV macrodomain (Fig. 4C,D), consistent with the *K*_D_ of 58 µM from ITC measurements. In contrast, addition of 10 µM poly-ADP-ribose (PAR) did not significantly alter the melting temperature of RuV macrodomain (Fig. 4E).

### The RuV macrodomain hydrolyzes both MAR and PAR adducts

**Fig. 5:**
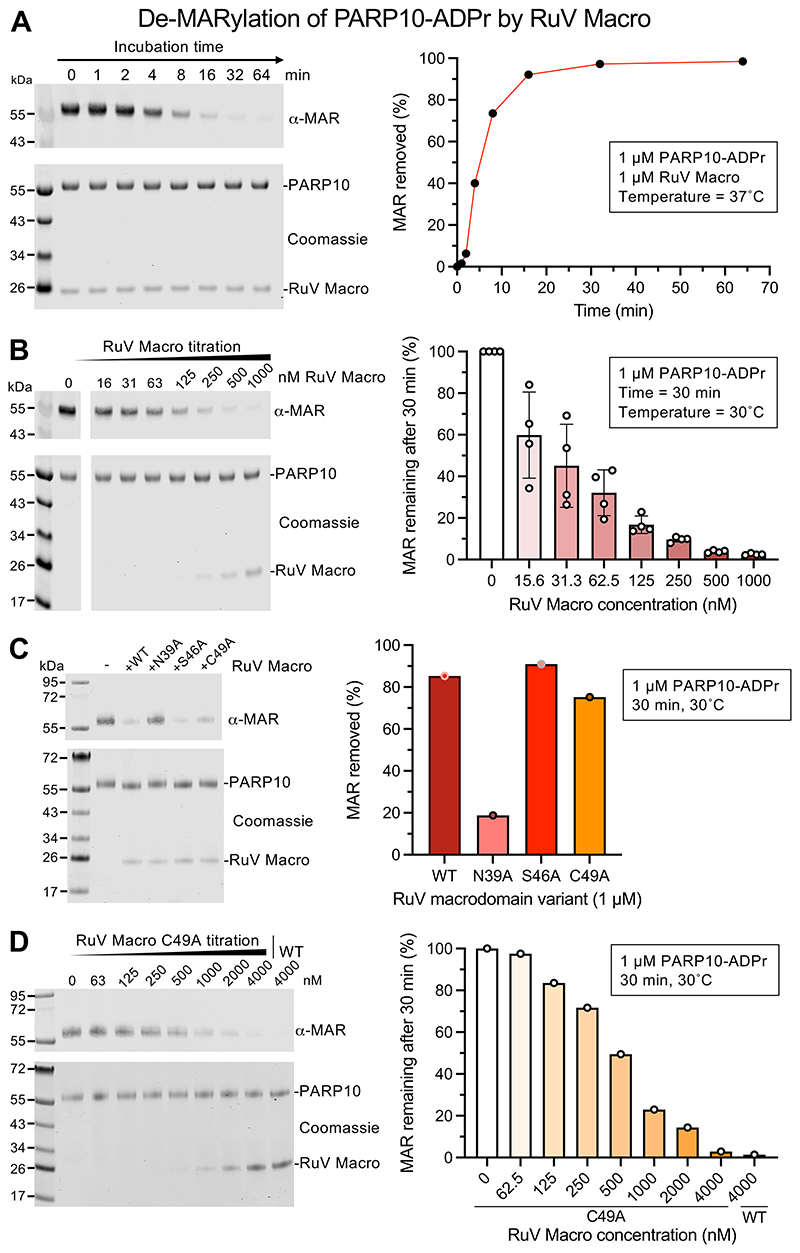
De-MARylation activities of wild type and mutant RuV macrodomains. (**A**) De-MARylation time course with wild type RuV macrodomain using MARylated PARP10 catalytic domain as the substrate. De-MARylation activity was measured as loss of signal in the anti-MAR Western blot (upper left). (**B**) De-MARylation of PARP10-ADPr as a function of RuV macrodomain concentration. (**C**) De-MARylation activities of RuV macrodomains with mutations in the ADPr binding site (N39A, S49A, and C49A), relative to wild type. (**D**) De-MARylation activity of the C49A RuV macrodomain mutant as a function of RuV macrodomain concentration.

To determine whether RuV macrodomain has mono-ADP-ribosylhydrolase (de-MARylase) activity, like other macroD-type structures, we performed a de-MARylation assay following a previously established protocol (23). Recombinant PARP10 catalytic domain was purified and incubated with NAD+ to allow it to MARylate itself. The ability of RuV macrodomain to de-MARylate auto-MARylated PARP10 was then assayed as a function of time and RuV macrodomain concentration. This de-MARylation assay clearly showed that RuV macrodomain was able to remove mono-ADP-ribosyl adducts from PARP10 (Fig. 5A,B). The extent, rate, and enzyme concentration dependence of PARP10 de-MARylation by RuV macrodomain were similar to those reported for SARS-CoV-2 Mac1 (23). Hence, RuV macrodomain is a catalytically active macroD-type macrodomain with de-MARylase activity.

To assess the relative contributions of substrate binding residues in RuV macrodomain we generated N39A, S46A, and C49A mutants and measured their de-MARylase activities. Consistent with the role of the second asparagine in the catalytic loop as a catalytic residue in other macroD proteins, the N39A mutation reduced the extent of de-MARylation 5-fold with an enzyme concentration of 1 µM (Fig. 5C). The C49A mutation only reduced the extent of de-MARylation by 11% at 1 µM of the enzyme (Fig. 5C) but the kinetics of de-MARylation were altered with a significant lag phase in hydrolysis compared to wild type RuV macrodomain (Fig. 5D). Hence, 500 nM of the C49A mutant was required to remove 50% of the MAR adducts from 1 µM MARylated PARP10 in 30 min, versus 20-25 nM for wild type RuV macrodomain (Fig. 5D). The S46A mutation did not affect de-MARylation activity, indicating that the side chain of serine 46 does not contribute to catalysis.

**Fig. 6:**
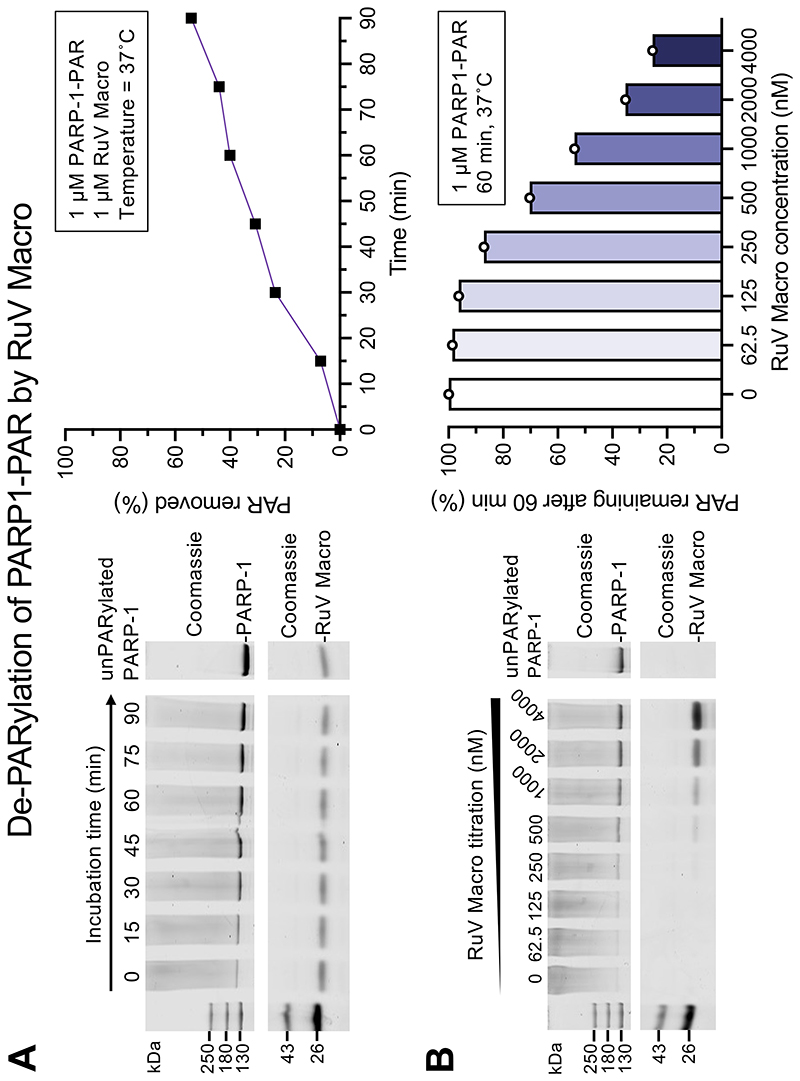
De-PARylation activity of RuV macrodomain. (**A**) De-PARylation time course with RuV macrodomain using PARylated full-length PARP1 as the substrate. De-PARylation activity was measured as gain of signal for unPARylated PARP1 in the Coommassie-stained SDS-PAGE gel (upper left). (**B**) De-PARylation of PARP1-PAR as a function of RuV macrodomain concentration.

Macrodomains from certain alpha- and coronaviruses, specifically Venezuelan equine encephalitis virus (VEEV) and SARS-CoV, were shown to hydrolyze poly-ADR ribose (PAR) adducts in addition to having de-MARylation activity (27). To determine whether RuV macrodomain could also hydrolyze PAR adducts, we performed a de-PARylation assay using PARylated full-length PARP-1 as the substrate. De-PARylation activity was detected in RuV macrodomain despite the absence of an active site glutamate residue, which is required for PAR hydrolysis by PARG-type macrodomains (Fig. 6). PAR hydrolysis was 16-fold slower than de-MARylation: RuV macrodomain took 80 min to de-PARylate 50% of the substrate, but only 5 min to de-MARylate 50% of the substrate, with the enzyme and substrate concentrations both at 1 µM (Figs. 5A and 6A).

## Discussion

We have shown here that the predicted macrodomain from rubella virus p150 (RuV macro) has a similar structure and active site to macroD-type mono-ADP-ribosylhydrolases (de-MARylases). The RuV macrodomain active site contains all of the macroD-type signatures except for a serine substitution (Ser46) in the first macroD motif (Fig. 3A). A detailed comparison with the available macrodomain structures shows that the active site composition and organization of RuV macrodomain are most similar to those of macrodomains from alphaviruses, and to a lesser extent coronaviruses. RuV macrodomain binds ADP-ribose in a similar manner to other macrodomains. Solution-based ligand binding assays show that ADP-ribose binds to a single specific site on RuV macrodomain, resulting in a significant thermal stabilization of the fold. Moreover, we have shown that RuV macrodomain has both de-MARylase and de-PARylase activities.

The RuV macrodomain active site contains all the hallmarks of a macroD-type de-MARylase and a few atypical features. The second asparagine in macroD motif, Asn39 in RuV macrodomain, is the most important residue for catalysis based on its position in the catalytic loop and the loss of de-MARylase activity observed in the N39A mutant. Asn39 forms a hydrogen bond with the distal ribose of the ADP-ribose, an interaction that is broadly conserved in macrodomains from different viral, bacterial, and vertebrate species. Our de-MARylase assays show that Cys49, in the catalytic loop and coordinating ADP-ribose, also plays a role in catalysis, with the C49A mutation markedly reducing the initial rate of catalysis. This cysteine residue is conserved in other rubiviruses, alphaviruses, and some bacterial macroD-type macrodomains. In the Getah virus macrodomain this cysteine forms a covalent adduct with ADP-ribose (22). Another atypical substitution in the catalytic loop of RuV macrodomain is Ser46. A serine in a similar position in Getah virus (Ser30) contributes to ADP-ribose binding (22). The S46A mutation did not affect the activity of RuV macrodomain in our de-MARylation assay. However, given the structural similarity of Ser46 and Ser30 in the RuV and Getah virus macrodomains, respectively, it remains possible that Ser46 contributes to binding of certain substrate of RuV macrodomain.

Unlike human and bacterial macroD-type macrodomains, which do not hydrolyze PAR chains, the macroD-type macrodomains from VEEV and SARS-CoV can efficiently de-PARylate PARP1 (27). We show here that RuV macrodomain can also hydrolyze PAR adducts, albeit at a significantly lower rate than MAR adducts. De-PARylation activity in viral macroD-type macrodomains is not universal. For example, the macrodomain of coronavirus HCoV2-229E is unable to hydrolyze PAR adducts (43). Although PAR glycohydrolase (PARG) activity generally requires a glutamate in the active site (14), the macrodomains of SARS-CoV, VEEV, and RuV can hydrolyze PAR adducts despite the lack of a glutamate or aspartate in their active site. In the absence of an active site carboxylate, PARG activity has been proposed to arise from a substrate-assisted mechanism, whereby the glutamate or aspartate of the PAR adduct in the substrate protein contributes to catalysis (13). In this mechanism, the acetyl group in glutamate or aspartate adduct activates the conserved structural water near the α-phosphate to nucleophilically attack the distal ribose C1’ atom (43). Consistent with this, structural analyses of macrodomains from alpha- and coronaviral macrodomains suggests that some viral macrodomains have evolved to bind PAR, thereby facilitating internal or external PAR cleavage, and conferring endo- or exo-glycohydrolase activity (26). Hence, we conclude that the low but clearly measurable de-PARylation activity of RuV macrodomain arises from substrate-assisted catalysis of PARylated protein substrates. Both PAR binding and hydrolytic activity are required for alphavirus replication (48).

Our work demonstrates that, like alpha- and coronaviruses, rubiviruses encode a structurally conserved macrodomain with variable mono- and poly-ADP-ribosylhydrolase activities in order to counteract ADR-ribosylation deposited by PARPs that are expressed upon interferon induction as part of in the host innate immune response. The macrodomains from rubi-, alpha-, and coronaviruses should therefore be considered as potential targets for antiviral therapeutics or combination therapies against rubella or infections with other viruses with similar macrodomains. The RuV macrodomain crystal structures reported here could guide future efforts to develop these new therapeutics.

## Materials and Methods

### Protein expression and purification

A gene encoding the macrodomain (residues 805-983) of p150 from rubella virus (RuV) strain RVi/Brooklyn.NY.USA/98/1B CRS (GenBank JN635282; UniProt G3M8F4) was cloned into the pET24a plasmid (Novagen) with a C-terminal hexahistidine purification (His_6_) tag. Expression plasmids for RuV macrodomain mutants (N39A, S46A, and C49A) were generated by site-directed mutagenesis. *Escherichia coli* BL21 (DE3) cells (New England BioLabs) were transformed with this expression construct and grown at 37˚C in 2×TY medium. At an optical density (OD_600_) of 0.6-0.8, the incubator temperature was lowered to 18˚C and protein expression was induced with 0.2 mM IPTG. After 16 h, the bacteria were pelleted (6,000 g for 15 min) and stored at -70˚C until required. The cell pellet was resuspended in Macro-Wash Buffer (50 mM Tris pH 7.4, 0.3 M NaCl, 20 mM imidazole) supplemented with cOmplete EDTA-free protease inhibitors (Roche) and 1:10,000 (v/v) Benzonase (Sigma) and lysed by sonication. The lysate was centrifuged at 40,000 g for 30 min and the supernatant was loaded onto a HisTrap HP column (Cytiva) equilibrated in Macro-Wash Buffer. The column was washed with 30 column volumes of Macro-Wash Buffer and the protein eluted with 50 mM Tris pH 7.4, 0.3 M NaCl, 0.25 M imidazole. The protein was further purified by size-exclusion chromatography with a HiLoad 26/600 Superdex 75 pg column (Cytiva) in 20 mM HEPES pH 8, 0.15 M NaCl.

For purification of the PARP10 catalytic domain (PARP10cd), a gene encoding PARP10 residues 818-1025 (UniProt Q53GL7) codon-optimized for *E. coli* was cloned into the first multiple cloning site of pRSFDuet (Novagen) with an N-terminal glutathione S-transferase (GST) affinity tag. *Escherichia coli* BL21 (DE3) cells (New England BioLabs) transformed with this expression plasmid were grown at 37˚C in 2×TY medium, induced with 0.2 mM IPTG at OD_600_ = 0.6-0.8, and incubated at 18˚C for 16-18h. The cells were pelleted and resuspended in PARP-Wash Buffer (50 mM Tris pH 8.0, 0.2 M NaCl, 0.1 mM EDTA, 10% glycerol, 1 mM DTT) supplemented with cOmplete EDTA-free protease inhibitors (Roche) and 1:10,000 (v/v) Benzonase (Sigma). Cells were lysed by sonication. The lysate was centrifuged at 40,000 g for 30 min and the supernatant loaded onto a GST-Trap 4B column (Cytiva) equilibrated in PARP-Wash Buffer. The column was washed with 30 column volumes of PARP-Wash Buffer and the protein eluted with PARP-Wash Buffer containing 25 mM reduced glutathione (GSH). The protein was further purified by size-exclusion chromatography on a Superdex S200 10/300 Increase column (Cytiva) in 20 mM HEPES pH 8.0, 0.2 M NaCl, 10% glycerol, and 0.5 mM tris(2-carboxyethyl)phosphine hydrochloride (TCEP).

Expression and purification of full-length His-tagged human PARP-1 followed a previously established protocol (49). *E. coli* Rosetta 2 (DE3) cells were transformed with a pET28 vector encoding PARP-1 and induced at 16˚C with IPTG. The protein was purified from the cell lysate by nickel-affinity, heparin affinity, and size-exclusion chromatography using HisTrap HP, HiTrap Heparin HP, and Superdex S200 10/300 Increase columns (Cytiva), respectively. The protein was flash-frozen and stored at -70˚C in 25 mM HEPES pH 8.0, 150 mM NaCl, 1 mM EDTA, 0.1 mM TCEP.

### Crystallographic structure determination

Crystals were grown at 18˚C by sitting drop vapor diffusion. The purified protein was concentrated to 9 g L^-1^ (452 µM) and mixed with an equal volume of reservoir solution optimized from the PEG/LiCl Grid Screen (Hampton Research): 14.4% PEG 6K, 1.26 M LiCl, 0.1 M Bicine pH 9. Crystals were cryoprotected in reservoir solution supplemented with 10% glycerol, and frozen in liquid nitrogen. For the ADP-ribose-bound structure, crystals were soaked in mother liquor supplemented with 2 mM ADP-ribose for 30-45 min prior to freezing. X-ray diffraction data were collected at 100 K at Diamond Light Source beamlines I03 and I04. Data were processed with autoPROC v1.0.5 (50), and STARANISO v1.0.4 (51). Molecular replacement was performed with MrBUMP (41) as implemented in CCP4 (42) using an atomic search model based on the human protein-proximal ADP-ribosyl-hydrolase MacroD2 structure (PDB 4IQY) (43). An initial model was built using AutoBuild in PHENIX v1.20.1 (52), manually completed with COOT (53) and iteratively refined with PHENIX. See Table 1 for crystallographic data collection, refinement, and validation statistics.

### Isothermal titration calorimetry (ITC)

Binding of RuV macrodomain to ADP-ribose was analyzed in 20 mM HEPES pH 8, 0.15 M NaCl at 25˚C, with an AutoiTC200 calorimeter (MicroCal). The sample cell was loaded with 0.2 ml of 100 µM RuV macrodomain and the titrant syringe with 2 mM ADP-ribose. 20 serial injections of 2 μl ADP-ribose were performed at 3 min intervals. The stirring speed was 1,000 rpm and the reference power was maintained at 6.06 μcal/s. The net heat absorption or release associated with each injection was calculated by subtracting the heat associated with the injection of ADP-ribose to buffer. Thermodynamic parameters were extracted from a curve fit to the data to a single-site model with Origin 7.0 (MicroCal). Experiments were performed in duplicate.

### Differential scanning fluorimetry (DSF)

10 µl samples of 25 µM or 50 µM RuV macrodomain in 20 mM HEPES pH 8, 0.15 M NaCl, with or without addition of up to 8 mM ADP-ribose or 10 µM poly-ADP-ribose (R&D Systems, Inc.), were loaded into glass capillaries (NanoTemper) by capillary action. Intrinsic protein fluorescence at 330 nm and 350 nm, F330 and F350, respectively, was measured from 15˚C to 95˚C with a ramp rate of 2˚C per minute with a Prometheus NT.48 nano-fluorimeter (NanoTemper). The melting temperatures (T_m_ values) were calculated with the accompanying software (NanoTemper) as the temperature at the peak of the first derivative of F350:F330 versus temperature.

### Enzymatic de-ADP-ribosylation assay

Following a previously established protocol (23), auto-mono-ADP-ribosylated PARP10cd was obtained by incubating 10 μM purified PARP10cd in 1 mM NAD+ for 20 min at 37˚C in Reaction Buffer (50 mM HEPES pH 8.0, 0.15 M NaCl, 0.2 mM TCEP, 0.02% NP-40). The de-ADP-ribosylation reaction was performed by incubating purified RuV macrodomain with mono-ADP-ribosylated PARP10cd at 37˚C in Reaction Buffer at equimolar ratios (1 μM). The reaction was terminated at 0, 1, 2, 4, 8, 16, 32 or 64 min by adding SDS-PAGE sample loading buffer and incubating at 95˚ for 5 min. Following separation SDS-PAGE on a 4-20% gradient gel, the proteins were transferred to a PVDF membrane and the de-MARylation activity determined by Western blotting. The membrane was blocked in 5% milk in PBS, 0.05% Tween-20. The membrane was incubated for 3 h at 22˚C in Anti-mono-ADP-Ribose Binding Reagent (Sigma, MABE1076, RRID:AB_2665469; 1:3,000 dilution) in place of a primary antibody. The secondary antibody was anti-rabbit immunoglobin (Cell Signaling Technology, 5151S, RRID:AB_10697505, DyLight® 800; dilution 1:10,000 dilution, 30 min at 22˚C). Blots were imaged with the near-infrared system of an Odyssey fluorescent scanner (LI-COR Biosciences) after washing with PBS and water.

### Enzymatic poly-ADP-ribose (PAR) hydrolysis assay

Following a previously established protocol (49), 0.56 µM purified PARP-1 was incubated for 10 min at room temperature with 0.56 µM 18-bp double-stranded DNA formed by annealing the following nucleotides: 5′ GGGTTGCGGCCGCTTGGG 3′, 5′ CCCAAGCGGCCGCAACCC 3′. PARylated PARP-1 was obtained by addition of 2.8 mM NAD+ and further incubation for 10 min at room temperature. The PARylation reaction was quenched with 11 mM EDTA. PAR hydrolysis activity was measured by incubating 1 μM purified RuV macrodomain with 1 μM PARylated PARP-1 at 37˚C in 50 mM HEPES pH 8.0, 0.15 M NaCl, 0.2 mM TCEP, 0.02% NP-40. The reaction was terminated at 0, 15, 30, 45, 60, 75, 90 min by adding SDS-PAGE sample loading buffer and incubating at 95˚ for 5 min. Following separation SDS-PAGE on a 4-20% gradient gel, the de-PARylation activity determined by measuring the band intensity for de-PARylated PARP-1.

## Figures and Tables

**Table 1:** Crystallographic data collection, refinement, and validation statistics

Data collection	p150 macro (apo)	p150 macro–ADP-ribose
Space group	*I* 2 2 2	*I* 2 2 2
Cell dimensions		
*a, b, c* (Å)	72.68 87.57 135.51	72.83 87.45 135.83
α, β, γ (°)	90, 90, 90	90, 90, 90
Resolution (Å)	64.1 - 1.72 (1.949 - 1.723)	73.5 - 1.59 (1.774 - 1.594)
Total reflections	123,141 (5,343)	132,356 (7,965)
Unique reflections	23,298 (1,165)	31,161 (1,560)
Multiplicity*^a^*	5.3 (4.6)	4.2 (5.1)
Completeness (ellipsoidal)*^a^*	91.3% (53.5%)	91.7% (68.7%)
Completeness (spherical)	50.6% (8.3%)	53.6% (9.9%)
*<I>* / σ(*I)*	9.9 (1.6)	12.8 (1.5)
Wilson *B-*factor	21.7	22.1
*R*_merge_	0.112 (1.03)	0.061 (0.934)
*R*_pim_	0.052 (0.514)	0.033 (0.454)
CC1/2	0.997 (0.613)	0.999 (0.621)
**Refinement and Validation**		
Resolution (Å)	43 - 1.72 (1.79 - 1.72)	68 - 1.59 (1.65 - 1.59)
No. reflections, working set	23,293 (38)	31,159 (223)
No. reflections, test set	1,183 (2)	1,569 (11)
*R*_work_	0.1932 (0.3173)	0.1897 (0.3872)
*R*_free_	0.2273 (0.0643)	0.2052 (0.4503)	
No. non-hydrogen atoms	2,993	3,136	
Protein	2,717	2,717	
ADP-ribose	-	114	
Chloride ions	3	3	
Water	273	344	
Average *B*-factors*^b^*			
Protein (Å^2^)	32	30	
ADP-ribose (Å^2^)	-	39	
Water (Å^2^)	33	35	
RMS*^c^* deviations			
Bond lengths (Å)	0.004	0.004	
Bond angles (°)	0.61	0.69	
Ramachandran plot			
Favored (%)	98.09	97.27	
Allowed (%)	1.91	2.73	
Outliers (%)	0	0	
Clashscore (Phenix 1.20.1)	5.72	7.23	
PDB accession code	8P0C	8P0E	

Numbers in parentheses refer to highest resolution shell.

a As defined in STARANISO (51).

b Residual B-factors after TLS refinement. See PDB entry for TLS parameters.

c RMS, root mean square

**Table 2:** Binding affinities of macrodomains to ADP-ribose.

Macrodomain	K_D(ADPr)_ (μM)	Method used	Reference
Rubella virus macro	58	ITC	This study
SARS-CoV-2 Mac1	11.4	ITC	(55)
	13	ITC	(56)
	15.5	FP	(57)
	44.5	MST	(48)
HCoV-229E macro	29	ITC	(58)
CHICKV macro	5.0	ITC	(25)
	15.4	FP	(57)
VEEV macro	3.9	ITC	(25)
	18.3	FP	(57)
SARS-CoV-1 macro	24	ITC	(59)
HEV macrodomain	>50	ITC	(59)
PARP14 MD2	7.8	ITC	(60)
AF1521	0.126	ITC	(12)

## Data Availability

The atomic coordinates of the rubella p150 macrodomain with and without ADP-ribose were deposited in the Protein Data Bank with accession codes 8P0C [https://doi.org/10.2210/pdb8p0c/pdb] and 8P0E [https://doi.org/10.2210/pdb8p0e/pdb].
